# Ethical Leadership as the Reliever of Frontline Service Employees’ Emotional Exhaustion: A Moderated Mediation Model

**DOI:** 10.3390/ijerph17030976

**Published:** 2020-02-04

**Authors:** Hao Zhou, Xinyi Sheng, Yulin He, Xiaoye Qian

**Affiliations:** Business School, Sichuan University, 29 Wangjiang Road, Chengdu 610064, China; zhouhao@scu.edu.cn (H.Z.); shengxinyi@stu.scu.edu.cn (X.S.); hyling_sc@stu.scu.edu.cn (Y.H.)

**Keywords:** emotional exhaustion, ethical leadership, organizational embeddedness, job satisfaction, conservation of resources theory

## Abstract

Based on the conservation of resources theory, this study aims to create new knowledge on the antecedents of emotional exhaustion. We explore the internal mechanism and boundary conditions of the impact of ethical leadership on emotional exhaustion, using data gathered from 460 frontline service employees at an airport in China. Employees completed questionnaires regarding ethical leadership, emotional exhaustion, organizational embeddedness, job satisfaction, and demographic variables. After controlling for the effects of demographic variables and company tenure, ethical leadership was found to have a negative impact on emotional exhaustion (β = −0.128, *p* < 0.01), and to be positively related to organizational embeddedness (β = 0.518, *p* < 0.01). After adding in the mediating variable (organizational embeddedness), the effect of ethical leadership on emotional exhaustion was no longer significant (β = 0.012, ns), while organizational embeddedness emerged as significantly related to emotional exhaustion (β = −0.269, *p* < 0.01), implying that the effect of ethical leadership on emotional exhaustion was completely mediated by organizational embeddedness. Simultaneously, the results suggested that job satisfaction could strengthen the mediating effect of organizational embeddedness on emotional exhaustion (the difference in the mediating effect between the groups with respective high and low job satisfaction was −0.096, *p* < 0.05). This study proposed and validated a moderated mediation model, the implications of which are that ethical leadership is an effective way to alleviate frontline service employees’ emotional exhaustion.

## 1. Introduction

Job burnout, defined as a state of exhaustion and cynicism towards work [1], has become an established focus of scholars’ attention in the last few decades. Some scholars describe burnout as an epidemic [2]. On May 28th, 2019, the World Health Organization announced that burnout had been included in the 11th Revision of International Classification of Diseases (ICD−11) as an occupational phenomenon [3]. Emotional exhaustion, the core dimension of burnout [4], is not only detrimental to employees’ physical and mental health, but is also harmful to enterprises’ sustainable development. According to previous studies, employees who become emotionally exhausted display sabotage behaviors, poor performance, high turnover intentions, and so on [5,6,7]. Unfortunately, emotional exhaustion is a widespread phenomenon, especially among frontline service employees. This is because frontline service employees have to regulate and maintain positive emotions in order to meet customers’ needs. Influenced by certain aspects of Chinese culture, such as high-power distance, Chinese frontline service employees undertake their duties following these normative requirements and endure any grievances that arise [8]. With an increase in pressure, their work not only drains their physical and mental energy, but also their emotional energy. By 2018, 44.6% Chinese employees worked in the services industry, according to the *2019 China Statistical Yearbook* [9]. China, the world’s most populous country, has an enormous number of frontline service employees. Therefore, it may be seen as meaningful for research to pay attention to these workers.

For employees, their supervisor acts as the agent of the organization and plays an important role in the former’s emotional exhaustion [10]. To date, ethical leadership has been proven to have an effect upon employees in areas such as task performance [11], voice behavior [12], helping behavior [13], etc. Given its significance in the current organizational context, this study introduces ethical leadership into the field of emotional exhaustion.

Conservation of resources theory (hereinafter referred to as COR theory) has been widely adopted across the field of research into burnout [14]. From the standpoint of COR theory, ethical leadership offers a kind of resource for employees. Organizational embeddedness on behalf of a state of resources overabundance [15,16]. With access to resources, frontline service employees may develop organizational embeddedness [17]. On the other hand, embedded employees have more resources [17] and can utilize the latter to alleviate emotional exhaustion. Thus, the current study took organizational embeddedness as a way to explore how ethical leadership affect frontline service employees’ emotional exhaustion.

Even if the active role of ethical leadership has been generally recognized, some studies have indicated that situational factors can influence employees’ perception of ethical leadership, meaning ethical leadership has different effects in different situations [18]. As a vital situational factor, job satisfaction may be seen as a positive emotional state, whereby an organization’s members evaluate their work or work environment [19]. Hence, the current study chose job satisfaction to discuss the boundary condition of the effects of ethical leadership on organizational embeddedness, and organizational embeddedness on emotional exhaustion.

Based on COR theory, this study creates a moderated mediation model to investigate how ethical leadership influences frontline service employees’ emotional exhaustion. Through this study, we provide a new organizational perspective for the alleviation of these employees’ emotional exhaustion. A key implication emerging from this research is that organizations should focus on the improvement of ethical leadership. With regard to organizational embeddedness, we further discuss the internal mechanism of how ethical leadership affects frontline service employees’ emotional exhaustion. These findings enrich the literature about the influential mechanisms of ethical leadership. Finally, job satisfaction was used to explore the boundary condition. When job satisfaction was high, ethical leadership was found to have a greater positive impact on organizational embeddedness. To better leverage the role of ethical leadership, this research signals that organizations need to focus on improving employees’ job satisfaction.

### 1.1. Ethical Leadership and Emotional Exhaustion

Ethical leadership not only demonstrates ethical behavior, but also inspires subordinates’ similar behavior by formulating rules, rewards, and punishments [18]. Previous studies have shown that supervisors’ ethical leadership has a positive impact on their subordinates [20,21]. For instance, such leadership can influence commitment, motivation, optimism, satisfaction, task performance, and organizational citizenship behavior [22,23,24,25]. Emotional exhaustion refers to a fatigued state due to the excessive consumption of psychological and emotional resources [26]. According to a previous study, emotional exhaustion can be influenced by situational factors [27]. Leadership is always an important situational factor in the organizational context [28] because subordinates always treat their supervisors as the organization’s agents. As a result of the increasing emphasis on social responsibility and business ethics, increasing numbers of researchers have begun to pay attention to the notion of ethical leadership [29]. Consistent with this trend, in the current paper we aim to analyze the impact of ethical leadership on frontline service employees’ emotional exhaustion and its mechanisms.

According to COR theory, people have a tendency to actively acquire, maintain, and protect what they think are valuable resources [30]. Ethical leadership can help frontline service employees gain access to more valued resources in the following ways. First, ethical leaders provide moral models (integrity, sincerity, etc.). This enables frontline service employees to have a trusting and optimistic view of their leaders and of the organization as a whole. Second, ethical leaders act in ways consistent with moral values. This can help frontline service employees to develop a long-term attachment to their leaders and organizations, which can effectively reduce their worries and uncertainty about the future. Third, ethical leaders encourage frontline service employees’ moral behavior and punish immoral behavior. Under such circumstances, frontline service employees will be friendly and respect each other. Thus, conflicts among colleagues are reduced and frontline service employees suffer less resource depletion. Fourth, ethical leaders treat frontline service employees fairly, pay attention to two-way communication, and help them solve work-related problems. This can stimulate the frontline service employees’ enthusiasm and vitality, in turn endowing them with the belief and ability required to access more valued resources. The development of burnout can be buffered by job resources [1]. Owning these resources can provide support for individual values and thus reduce emotional exhaustion [31]. In short, frontline service employees regard ethical leadership as a kind of resource, meaning that it helps to reduce emotional exhaustion. Hence, we put forward the following hypothesis:

**Hypothesis** **1**. *Ethical leadership will be negatively associated with emotional exhaustion*.

### 1.2. The Mediating Effect of Organizational Embeddedness

Lee, et al. [32] define job embeddedness as an extent of an employee’s “stuckness”, or enmeshing, resulting from numerous forces. Among the collection of forces which prevent individuals from leaving, job embeddedness can be divided into two aspects: organizational embeddedness and community embeddedness [33]. Due to the organizational context being the research focus at hand, we focused on organizational embeddedness in this research. Mitchell, et al. [34] proposed that organizational embeddedness included three sub-dimensions: fit, link, and sacrifice. ‘Fit’ refers to the compatibility between employees and organizations with regard to skills and values. ‘Link’ includes formal and informal connections between employees, colleagues and the organization as a whole. ‘Sacrifice’ refers to what employees have to give up when they leave the organization. As mentioned earlier, ethical leadership is proposed to have a negative effect on emotional exhaustion. We further propose the mediating role of organizational embeddedness.

Based on COR theory, the resources that frontline service employees receive from ethical leadership [35] can lead to an increase in organizational embeddedness. Specifically, ethical leaders guide and motivate frontline service employees to work towards organizational goals and to understand and identify with organizational values. Thus, ethical leadership enhances the compatibility between frontline service employees and their organizations, i.e., fit. Ethical leaders also promote altruistic behavior by acting as role models and striving to create a harmonious organizational climate. In such an atmosphere, frontline service employees become more cooperative and devoted to the organization—in other words, the link factor is strengthened. In addition, ethical leaders can also be reliable leaders who provide sustainable resources. Therefore, for frontline service employees, leaving would incur a loss, that is, a sacrifice. In short, ethical leadership can promote employee’s organizational embeddedness through improving the level of fit, link, and sacrifice [34]. When frontline service employees have a higher level of organizational embeddedness, they have access to more useful and supportive resources. This is because, with a high level of organizational embeddedness, they are more likely to identify with the organizational mission, have a high investment in teamwork and frequent interaction relationships, and present a positive performance. It is conducive for frontline service employees to better complete their work tasks and protect their emotional resources, which can, in turn, lead to them suffering less emotional exhaustion, which is a negative outcome caused by resource depletion [36].

From the perspective of resource acquisition, the resources that frontline service employees acquire from ethical leadership enhance their organizational embeddedness. From the perspective of resource utilization, organizational embeddedness alleviates employees’ emotional exhaustion which is caused by resource depletion. To summarize, organizational embeddedness reflects the two processes of resource acquisition and resource utilization, and reflects the influence of ethical leadership on emotional exhaustion [37]. Therefore, we predict the following:

**Hypothesis** **2**. *Organizational embeddedness will mediate the negative relationship between ethical leadership and emotional exhaustion*.

### 1.3. The Moderating Effects of Job Satisfaction

In spite of the positive effect of ethical leadership being generally recognized, some scholars have pointed out that situational factors will affect the effects of ethical leadership [18]. For example, injustice will weaken the positive effect of ethical leadership on employees [38]. Leader authenticity will interfere with employees’ positive judgment of ethical leadership [39]. Job satisfaction, an important boundary condition, refers to a positive emotional state relating to how an organization’s members evaluate their work or work environment [19]. On one hand, job satisfaction can be seen as influencing frontline service employees’ rating of ethical leadership. On the other hand, job satisfaction can be regarded as a kind of resource [19,31].

When frontline service employees experience a high level of job satisfaction, their sense of identity and trust in leaders will be enhanced. They can fully understand ethical leadership and regard ethical leaders as their role models. Thus, motivated by the recognition of ethical leadership, frontline service employees can increase their connection to the organizations and experience a higher degree of organizational embeddedness. On the contrary, frontline service employees who have a low level of job satisfaction are suspicious of their organization and leaders. Under these circumstances, even if ethical leaders exhibit positive behavior, such employees will scarcely feel good will, and are unable to rely on leaders. These employees would, therefore, be unwilling to further integrate into the organizations. On the basis of the above analysis, we posit the following:

**Hypothesis** **3a**. *Job satisfaction will moderate the positive relationship between ethical leadership and organizational embeddedness, such that this relationship will be stronger when job satisfaction is high and weaker when job satisfaction is low*.

According to COR theory, frontline service employees with more resources will be more resilient to stress. Job satisfaction may also be seen as a kind of resource in this context [19,31]. Frontline service employees with a high degree of job satisfaction tend to obtain more supportive resources owing to their organizational embeddedness. When frontline service employees have more resources with which to respond to job demands, they experience less emotional exhaustion. On the contrary, in order to protect their resources, frontline service employees experiencing low job satisfaction will take action to prevent further resource depletion [31,40]. Thus, they have fewer available resources to deal with emotional exhaustion. Simultaneously, the effect of organizational embeddedness on emotional exhaustion will be weakened. We thus hypothesize the following:

**Hypothesis** **3b**. *Job satisfaction will moderate the negative relationship between organizational embeddedness and emotional exhaustion, such that this relationship will be stronger when job satisfaction is high and weaker when job satisfaction is low.*

Combining the above analyses, frontline service employees with a higher degree of job satisfaction have a clearer understanding of ethical leadership and are more inclined to regard ethical leaders as a reliable source of resources, resulting in these employees’ higher organizational embeddedness. At the same time, frontline service employees with higher job satisfaction are more active in their work and more deeply embedded in their organization. They are good at using organizational resources to respond to work needs, which results in less emotional exhaustion. Therefore, the higher the level of employees’ job satisfaction, the stronger the mediating role of organizational embeddedness in the relationship between ethical leadership and employees’ emotional exhaustion. As such, we put forward the following, final hypothesis:

**Hypothesis** **3c**. *Job satisfaction will moderate the indirect effect of ethical leadership on emotional exhaustion via organizational embeddedness, such that the indirect effect will be stronger when job satisfaction is high.*

**Figure 1:**
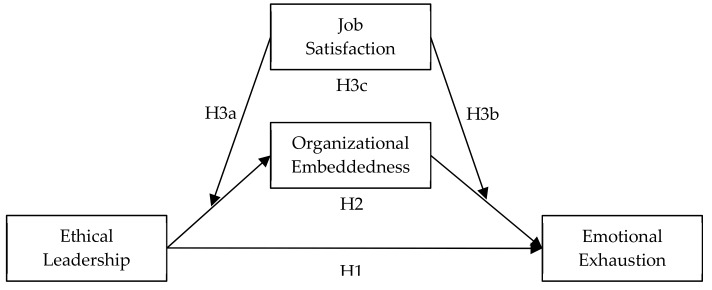
Theoretical model

In summary, we propose a moderated mediation model, as shown in Figure 1.

## 2. Materials and Methods

### 2.1. Participants and Procudures

Data were collected at an airport in the southwest of China using structured questionnaires. We sought to obtain a 0.05 margin error with a 95% confidence level, which required 384 valid samples. Thus, in order to obtain sufficient valid samples, we distributed questionnaires to 500 airport frontline service employees. Following the principle of convenience, we randomly selected samples in each frontline service department. Researchers adopted the data collection methods of on-site distribution and on-site recovery. Participants voluntarily engaged and answered the anonymous, self-administered questionnaire. The questionnaires were sent out with envelopes, whereby participants returned the completed questionnaires in sealed envelopes. We also confirmed that the information they provided was intended for research purposes only.

### 2.2. Measures

In this study, we used the following, well-established scales and translated them into Chinese according to the procedure of translation and back-translation [41]. Prior to the formal data collection, we invited several frontline service employees to participate in a pilot survey using the intended questionnaire. They reported that there was no confusion or misunderstanding in the questionnaire.

Ethical leadership. Considering China’s cultural background, we adopted eight items from the Brown, Treviño and Harrison [18] scale to measure ethical leadership. A 5-point Likert scale (1 = strongly disagree; 5 = strongly agree) was employed. A sample item here was as follows: “My supervisor listens to what employees have to say”. Cronbach’s α was 0.974.

Emotional exhaustion. We assessed emotional exhaustion using the three-item scale developed by Watkins, et al. [42]. Participants responded to a 5-point Likert scale (1 = strongly disagree; 5 = strongly agree). A sample item here was: “I feel emotionally drained by my work”. Cronbach’s α was 0.901.

Organizational embeddedness. Participants responded to three items developed by Cunningham, et al. [43] to assess organizational embeddedness. A 7-point Likert scale (1 = strongly disagree; 7 = strongly agree) was used to measure participants’ responses. A sample item here was as follows: “I feel compatible with my organization”. Cronbach’s α was 0.833.

Job satisfaction. We assessed job satisfaction using the three-item scale developed by Hackman and Oldham [44]. Items were anchored by a 5-point Likert scale (1 = strongly disagree; 5 = strongly agree). A sample item here was as follows: “Generally speaking, I am very satisfied with this job”. Cronbach’s α was 0.884.

Control variables. Gender, age, education and company tenure were applied as control variables.

### 2.3. Statistical Analysis

We ran a series of confirmatory factor analyses (CFAs) and tested common method biases for preliminary analyses. SPSS 22.0 was employed to validate our hypotheses. We tested the mediating effect of organizational embeddedness following the procedure proposed by Baron and Kenny [44]. We examined the moderating effect of job satisfaction with reference to the general analytical framework proposed by Edwards and Lambert [45].

### 2.4. Ethical Statement

According to the particular institutional guidelines and national laws and regulations, no ethical approval was required for our research, because our study did not involve human clinical trials or animal experiments. We took steps to ensure that participants’ information was kept secure and private. In addition, all of the frontline service employees who participated did so on a voluntary basis. The oral consent of each of these participants was obtained before the study began.

## 3. Results

### 3.1. Participant Characteristics

460 of the 500 participants submitted valid questionnaires, resulting in a response rate of 92 percent. The average age of the frontline service employees was 33.58; the average company tenure was 56.79 months. There were 135 women (29.3%) and 325 men (70.7%). The most frequently reported education levels were “high school” (63%) and “college” (28.7%).

### 3.2. Confirmatory Factor Analyses and Test of Common Method Bias

We conducted a series of confirmatory factor analyses to test the distinctiveness of the constructs. The results of the confirmatory factor analyses are presented in Table 1. The results indicate that the 4-factor model (ethical leadership, organizational embeddedness, emotional exhaustion, and job satisfaction) fitted the data better than the other models. Thus, the distinctiveness of the four measures was confirmed.

As all of the measures in the questionnaire we designed were self-reported, we performed Harman’s single-factor test to examine any common method biases [45]. As shown in Table 1, the single factor model had a poor fit with the data. It can thus be concluded that there were no significant common method biases in our measurement.

### 3.3. Descriptive Statistics and Correlations

Means, standard deviations, and zero-order correlations among the study variables are displayed in Table 2. Here, ethical leadership can be seen to be negatively related to emotional exhaustion (r = −0.099, *p* < 0.05), and positively associated with organizational embeddedness (r = 0.526, *p* < 0.01). At the same time, organizational embeddedness has a negative effect on emotional exhaustion (r = −0.255, *p* < 0.01). These results provide initial support for the research hypotheses.

### 3.4. Hypotheses Testing

We tested our hypotheses in three steps, following the procedure developed by Baron and Kenny [46]. Firstly, we examined the effect of ethical leadership on emotional exhaustion. As shown in Model 2 of Table 3, ethical leadership emerged as negatively related to emotional exhaustion (β = −0.128, *p* < 0.01), after controlling for the effects of gender, age, education, and company tenure. Hypothesis 1 was thus verified. Secondly, we examined the effect of ethical leadership on organizational embeddedness. As shown in Model 5 of Table 3, ethical leadership was found to be positively related to organizational embeddedness (β = 0.518, *p* < 0.01). Thirdly, we tested the effect of organizational embeddedness on emotional exhaustion. As shown in Model 3 of Table 3, organizational embeddedness was negatively related to emotional exhaustion (β = −0.269, *p* < 0.01), whereas ethical leadership had no significant effect on the latter (β = 0.012, ns). Above all, the effect of ethical leadership on emotional exhaustion was found to be totally mediated by organizational embeddedness, thus supporting Hypothesis 2.

**Figure 2:**
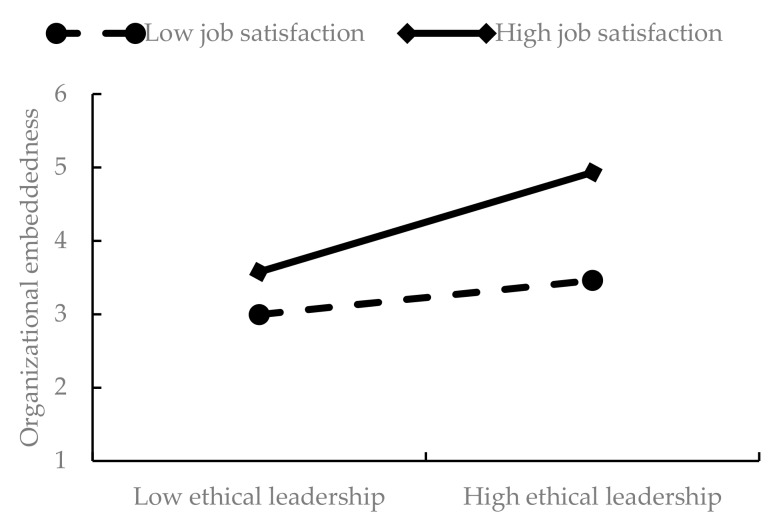
Moderating effect of job satisfaction on the relationship between ethical leadership and organizational embeddedness.

To examine the moderating effect of job satisfaction, we adopted the general analytical framework proposed by Edwards and Lambert [47]. For the first stage of the proposed model (the relationship between ethical leadership and organizational embeddedness), the difference between the groups experiencing high job satisfaction and low job satisfaction was significant (0.820−0.533 = 0.287, *p* < 0.01, respectively), as shown in Table 4. Hypothesis 3a was thus supported. As shown in Figure 2, the relationship between ethical leadership and organizational embeddedness was stronger for those who felt they had a high level of job satisfaction.

However, as shown in Table 4, for the second stage of the proposed model (the relationship between organizational embeddedness and emotional exhaustion), the difference between the group experiencing high job satisfaction and that with low job satisfaction was not significant (−0.204 – (−0.133) = −0.071, ns). Hypothesis 3b was thus not supported.

Hypothesis 3c proposed that job satisfaction would moderate the indirect effect of ethical leadership on emotional exhaustion via organizational embeddedness. As shown in Table 4, there was a significant difference in the indirect effect between the groups with respective high and low job satisfaction (−0.167 – (−0.071) = −0.096, *p* < 0.05); as previously predicted, the indirect effect was stronger when the reported level of job satisfaction was high, thus supporting Hypothesis 3c.

In conclusion, these results show that ethical leadership had a positive effect on these frontline service employees’ state of emotional exhaustion. Organizational embeddedness played a mediating role in the relationship between ethical leadership and emotional exhaustion. The moderating role of job satisfaction was also supported. The validation of our moderated mediation model provides a new perspective from which to engage with frontline service employees.

## 4. Discussion

### 4.1. Theoretical Contributions

Emotional exhaustion, as a health problem, is increasing around the world [48]. Such exhaustion is strongly associated with depression. Moreover, frontline service employees’ emotional exhaustion has been seen consistently to lead to poor customer service performance [49]. In the current study, it was thus deemed valuable to explore methods to reduce frontline service employees’ emotional exhaustion from an organizational perspective

First, in this research, we associated ethical leadership with frontline service employees’ emotional exhaustion, and the results showed that ethical leadership had a negative effect on such emotional exhaustion. Our research extends the study of ethical leadership into the field of emotion and enriches the research literature pertaining to ethical leadership and emotional exhaustion. Specifically, the results of our study support the previously indicated negative effect of ethical leadership on emotional exhaustion [50], and prove that this effect is also significant for frontline service employees. Furthermore, the current study provides a new perspective for analyzing the causes of frontline service employees’ emotional exhaustion. Ethical leadership not only establishes an organization’s basic ethical norms, but also means that higher ethical standards are adhered to at management level. This, in turn, provides more resources for frontline service employees and thus helps to reduce their emotional exhaustion.

Second, our research explains the internal mechanism of the relationship between ethical leadership and emotional exhaustion, based on COR theory. Employees obtain supportive resources from ethical leaders [35] in order to enhance their organizational embeddedness, reflecting a form of resource acquisition. Employees with a high-level of job embeddedness are better able to cope with workplace stress and reduce emotional exhaustion [14], reflecting a better utilization of resources. This study provides a new perspective from which to construe the effect of ethical leadership on subordinates’ psychology and behavior, and enriches the literature on the influential potential of ethical leadership.

Third, our research further explores the boundary conditions of the impact of ethical leadership by verifying the moderating role of job satisfaction. Specifically, job satisfaction was found to have a significant moderating effect on the relationship between ethical leadership and organizational embeddedness, and also on the mediating effect of ethical leadership on emotional exhaustion via organizational embeddedness. Certain factors can act as “substitutes for leadership” and influence the effect of leadership [51]. Subordinates tend to combine managers’ moral performance with contextual clues in organizations [52,53]. If the two are consistent, the impact of ethical leadership will be magnified. Otherwise, managers’ ethical leadership will be regarded as affectation and hypocrisy. In this way, ethical leadership can have a less positive effect, or even exert a negative influence. Overall, our study provides a theoretical reference for further understanding the influential effect of ethical leadership.

We proposed that job satisfaction would have a moderating effect on the relationship between organizational embeddedness and emotional exhaustion, but the results did not support this. However, we found that job satisfaction was negatively related to emotional exhaustion (r = −0.247, *p* < 0.01). This suggests that, when an employee’s resources are used to cope with emotional exhaustion, different resources cannot be substituted for each other. This finding supports the previous idiographic approaches, which describe resource value in terms of one’s demand for those resources [37]. Employees will distinguish between the sources of different resources. In other words, from the perspective of such sources, our research provides new knowledge regarding COR theory.

### 4.2. Practical Implications

Frontline service employees’ emotional exhaustion will result in low efficiency and great economic loss [54]. The findings of the current study indicate that ethical leadership is an effective method of reducing emotional exhaustion. The moral model set by ethical leadership can alleviate and eliminate frontline service employees’ emotional exhaustion. In light of these findings, it may be seen as necessary for enterprises to improve their level of ethical leadership. How is this to be accomplished? There are several dimensions for organizations to consider. First, organizations should pay attention to the examination of managers’ moral character and try to select employees with a higher level of morality for promotion. They should also establish and improve the reward and punishment mechanism for ethical behavior and reward ethical leadership. Second, organizations can provide training and communication to enhance managers’ ethical characteristics and related skills. Third, organizations can cultivate an ethical culture and create a good atmosphere. With regard to managers themselves, it is recommended that they consistently work to improve their moral cultivation, set an example, pay attention to maintaining communication and interaction with their subordinates, and strive to create a good moral atmosphere in the organization.

This study found a mediating effect of organizational embeddedness on the relationship between ethical leadership and emotional exhaustion, suggesting that organizational embeddedness is a proximal factor affecting emotional exhaustion. Organizations and managers can reduce frontline service employees’ emotional exhaustion by improving organizational embeddedness, which can be undertaken in the following ways. According to Mitchell, Holtom, Lee, Sablynski and Erez [34], organizational embeddedness can be improved in three ways, namely, by promoting the aspects of fit, link and sacrifice. This is done by selecting frontline services employees who are aligned with the organization’s values. After entry, organizations can train frontline services employees in such a way as to enhance the fit between them and the organization. Secondly, organizations and managers need to attach importance to frontline service employees’ interpersonal interactions in the organization, because good interpersonal interactions can enhance communication between employees, improve frontline service employees’ trust and commitment to the organization, and thereby strengthen the connection between frontline service employees and their organization. Finally, the organization should not only provide a good working environment and financial resources for frontline service employees, but should also provide care, respect, and moral examples. In this way, organizations can increase the frontline service employees’ perceived cost of turnover.

This study finds that job satisfaction is an important boundary condition. In addition to their own positive moral performance, the results indicate that moral leaders can also use favorable situational clues to enhance the effectiveness of ethical leadership. Specifically, leadership behavior is a process of interaction among managers, subordinates, and situations. It is necessary for ethical leaders to create a harmonious democratic atmosphere, attach importance to two-way communication with subordinates, care for subordinates, and build a community of common interests with the same goal. Ethical leaders can help frontline service employees manage their career, improve job satisfaction [55,56] and, ultimately, reduce their emotional exhaustion.

### 4.3. Limitations and Directions for Future Research

Firstly, this study introduced organizational embeddedness as a mediating variable to explain how ethical leadership affects emotional exhaustion. Future studies are needed to test the applicability of our theoretical model and expand this to other psychological and behavioral variables. Secondly, we collected data from airport frontline service employees; whether the results can be applied to other industries needs further testing. Finally, this study adopted a cross-sectional design. Future studies would benefit from applying a longitudinal or experimental design to explore the causal relationship among ethical leadership, organizational embeddedness and emotional exhaustion.

## 5. Conclusions

Based on COR theory, this study proposed and tested a theoretical model of the relationship between ethical leadership, organizational embeddedness, job satisfaction, and emotional exhaustion. Following an analysis of data gathered from frontline service employees at an airport, the results confirmed a negative, indirect effect between ethical leadership and emotional exhaustion via organizational embeddedness, and the moderating effect of job satisfaction. Based on this, it can be concluded that ethical leadership is an effective way to alleviate frontline service employees’ emotional exhaustion. Future research could explore ways of alleviating employees’ emotional exhaustion from the perspective of organizational psychology.

## Figures and Tables

**Table 1:** Results of confirmatory factor analyses models.

model	*χ^2^*	*df*	*χ^2^/df*	*RMSEA*	*TLI*	*CFI*
4-factor model	497.785	113	4.405	0.086	0.941	0.951
3-factor model a	927.655	116	7.997	0.123	0.879	0.897
3-factor model b	1434.223	116	12.364	0.157	0.804	0.833
Single-factor model	2351.907	119	19.764	0.202	0.676	0.717

Note. “3-factor model a” combines ethical leadership and organizational embeddedness. “3-factor model b” combines organizational embeddedness and emotional exhaustion.

**Table 2:** Means, standard deviations, and correlations.

	*M*	*SD*	1	2	3	4	5	6	7	8
1. Gender	1.29	0.456	--							
2. Age	33.58	8.186	0.144 **	--						
3. Education	1.46	0.664	–0.316 **	–0.522 **	--					
4. Tenure (month)	56.79	49.359	–0.014	0.645 **	–0.237 **	--				
5. Ethical leadership	4.08	0.942	0.171**	–0.100 *	0.047	–0.072	(0.974)			
6. Emotional exhaustion	2.94	1.212	0.218**	0.127 **	–0.166 **	0.051	–0.099 *	(0.901)		
7. Organizational embeddedness	4.74	1.275	0.080	–0.092 *	0.137 **	–0.067	0.526 **	–0.255 **	(0.833)	
8. Job satisfaction	3.59	0.795	–0.071	–0.061	0.098 *	–0.022	0.566 **	–0.247 **	0.573 **	(0.884)

Note. Gender: 1 = male, 2 = female. Education: 1= “graduated from technical secondary school or blew”, 2 = “graduated from junior college”, 3= “had a college degree or above”. * *p* < 0.05, ** *p* < 0.01. N = 460.

**Table 3:** Results of mediating effect test.

	Emotional Exhaustion			Organizational Embeddedness	
	Model 1	Model 2	Model 3	Model 4	Model 5
Gender	0.185 **	0.211 **	0.219 **	0.135 **	0.029
Age	0.066	0.052	0.064	−0.012	0.044
Education	−0.074	−0.067	−0.029	0.169 **	0.139 **
Tenure	−0.006	−0.005	−0.011	−0.017	−0.024
Ethical leadership		−0.128**	0.012		0.518 **
Organizational embeddedness			−0.269 **		
R^2^	0.061	0.076	0.127	0.036	0.291
ΔR^2^	0.061 **	0.015 **	0.051 **	0.036 **	0.255 **
F	7.338 **	7.069 **	26.683 **	4.282 **	163.480 **

Note. N = 460. * *p* < 0.05, ** *p* < 0.01.

**Table 4:** Results of moderated mediation effect.

	Stage		Effect		
	First Stage	Second Stage	Direct Effect	Indirect Effect	Total Effect
High job satisfaction (4.3843)	0.820 **	−0.204 **	−0.137	−0.167 **	−0.304 *
Low job satisfaction (2.7939)	0.533 **	−0.133	0.043	−0.071	−0.028
Inter-group difference	0.287 **	−0.071	−0.180	−0.096 *	−0.276 **

Note. N = 460, * *p* < 0.05, ** *p* < 0.01. Job satisfaction_high_ = mean + 1sd, Job satisfaction_low_ = mean−1sd, bootstrap sample = 1000.
